# Supplementary material for: MetaRibo-Seq measures translation in microbiomes
Source: Nat Commun. 2020 Jun 29;11:3268. doi: 10.1038/s41467-020-17081-z (PMC7324362; doi:10.1038/s41467-020-17081-z)
Supplement: Supplementary file 10 — Supplementary Data 7 [file 41467_2020_17081_MOESM10_ESM.zip › File2/Confidence_High_Taxonomy/136833_out.krona.html]

Javascript must be enabled to view this page.

members
magnitude
magnitudeUnassigned
count
unassigned
taxon
rank

136833\_out

13

13
2
superkingdom

phylum
1239
13

186801
13
class

order
186802
13

1
424536
family

270497
1
genus

species
1

SRS016056\_contig\_number\_449
1946296

family
186803
3

3
572511

SRS017821\_contig\_number\_9710SRS023715\_contig\_number\_contig-100\_16816.137620SRS147346\_contig\_number\_contig-100\_29253.88797
genus

1
31979
family

1
1485
genus

species

SRS062427\_contig\_number\_contig-100\_8101.48082
2292307
1

8
541000
family

genus
1263
8

species
165186

SRS015217\_contig\_number\_13578SRS021153\_contig\_number\_contig-100\_8084.43099SRS057717\_contig\_number\_contig-100\_6786.33377SRS146888\_contig\_number\_contig-100\_9007.81259SRS148511\_contig\_number\_contig-100\_18880.66644SRS148721\_contig\_number\_23074SRS149879\_contig\_number\_20712SRS893256\_contig\_number\_contig-100\_18290.18291
8
